# 3D Printing in Otolaryngology Surgery: Descriptive Review of Literature to Define the State of the Art

**DOI:** 10.3390/healthcare11010108

**Published:** 2022-12-29

**Authors:** Federica Zoccali, Andrea Colizza, Fabrizio Cialente, Arianna Di Stadio, Ignazio La Mantia, Charlie Hanna, Antonio Minni, Massimo Ralli, Antonio Greco, Marco de Vincentiis

**Affiliations:** 1 Department of Sense Organs, Sapienza University of Rome, 00161 Rome, Italy; 2 Department GF Ingrassia, University of Catania, Via di Santa Sofia 78, 95125 Catania, Italy; 3 Faculty of Medicine and Medical Sciences, Holy Spirit University of Kaslik (USEK), Jounieh 1200, Lebanon; 4 Department of Otolaryngology Head and Neck Surgery, Eye and Ear International Hospital, Naccache 1201, Lebanon

**Keywords:** 3D printing, bioprinting, otorhinolaryngology-ENT surgery applications, rapid prototyping

## Abstract

Background: Three-dimensional (3D) printing has allowed great progression in the medical field. In otolaryngology practice, 3D printing can be used for planning in case of malformation/complex surgery, for surgeon training, and for recreating missing tissues. This systematic review aimed to summarize the current benefits and the possible future application of 3D technologies in the otolaryngology field. Methods: A systematic review of articles that discuss the use of 3D printing in the otolaryngology field was performed. All publications without the restriction of time and that were published by December 2021 in the English language were included. Searches were performed in the PubMed, MEDLINE, Scopus, and Embase databases. Keywords used were: “3D printing”, “bioprinting”, “three-dimensional printing”, “tissue engineering” in combination with the terms: “head and neck surgery”, “head and neck reconstruction”, “otology”, “rhinology”, “laryngology”, and “otolaryngology”. Results: Ninety-one articles were included in this systematic review. The articles describe the clinical application of 3D printing in different fields of otolaryngology, from otology to pediatric otolaryngology. The main uses of 3D printing technology discussed in the articles included in the review were surgical planning in temporal bone malformation, the reconstruction of missing body parts after oncologic surgery, allowing for medical training, and providing better information to patients. Conclusion: The use of 3D printing in otolaryngology practice is constantly growing. However, available evidence is still limited, and further studies are needed to better evaluate the benefits of this technology.

## 1. Introduction

The first 3D printing (3DP) technology able to create several 3D objects starting from digital data was developed by Hull et al. in 1986 [1]; the system could create 3D objects with information from digital databases [2]. Three-dimensional printing allows us to recreate an object in three dimensions and is extremely important in medical imaging [3,4]; for example, 3D Computer Tomography (CT) represents the gold standard investigation in maxillofacial trauma to obtain the best functional and aesthetic results after surgery [3,4]. Recently, 3DP has been used in different medical and surgical disciplines including otolaryngology, maxillofacial surgery, orthopedics, neurosurgery, and cardiac surgery. It is also specifically important in precision medicine and for research purposes [5].

In the otolaryngology field, 3DP has been mainly used to create hand-held anatomical models, biologic tissue scaffolds, personalized patient-specific implants, surgical planning in difficult cases, and surgical training [6,7]. The first clinical application of 3DP in otolaryngology was the resection and reconstruction of oromandibular tumors, followed by the introduction of this technology in other areas such as otology and rhinology.

This systematic review of the literature aimed at describing the state of the art of this new technology in the otolaryngology field.

## 2. Materials and Methods

### 2.1. Literature Search Strategy

After conducting an initial background research to define the conceptual foundations of this study, a systematic literature review was performed as recommended in the Preferred Reporting Items for Systematic and Meta-Analyses (PRISMA) statement and was guided by the associated checklist. Two independent researchers (FZ and FC) performed a review of the scientific literature using the following international databases: PubMed, MEDLINE, Embase, and Scopus.

The keywords used were: “3D printing”, “bioprinting”, “three-dimensional printing”, “tissue engineering” in combination with the following terms: “head and neck surgery”, “head and neck reconstruction”, “otology”, “rhinology”, “laryngology”, and “otolaryngology”. The researchers screened the databases using the keywords and a combination of them, with the “3D printing” or “three-dimensional printing” terms always included in the research string.

### 2.2. Study Selection

The systematic review of the literature was conducted from January to March 2022 with no time restrictions by two independent researchers (FZ and FC).

Studies had to meet the following criteria to be included in the review: (a) articles in the English language; (b) studies conducted on human subjects (adults and children); (c) 3DP used in the otolaryngology field. Exclusion criteria were: (a) studies with poor surgical or clinical relevance (letters and commentaries); (b) engineering studies; (c) articles that discussed 3DP applications that do not exclusively belong to the otolaryngology field (e.g., use of 3DP for maxillofacial surgery).

The title and abstract were examined by two authors independently (FZ and FC), and disagreements were resolved by a discussion with a third author (AC).

### 2.3. Data Extraction

Once the articles that fit the inclusion criteria were identified, each researcher (FZ, FC, and AC) read in full each article and added it to a digital database (Microsoft Excel, Microsoft Inc., Redmond, WA, USA).

The database included author names, year of publication, type of study, number of patients included, aims, interventions and outcomes, impact on treatment time, level of validity of the printed part, and clinical outcome.

All the studies included were categorized by the specific field of interest such as otology, rhinology, laryngology, and pediatrics.

## 3. Results

**Figure 1:**
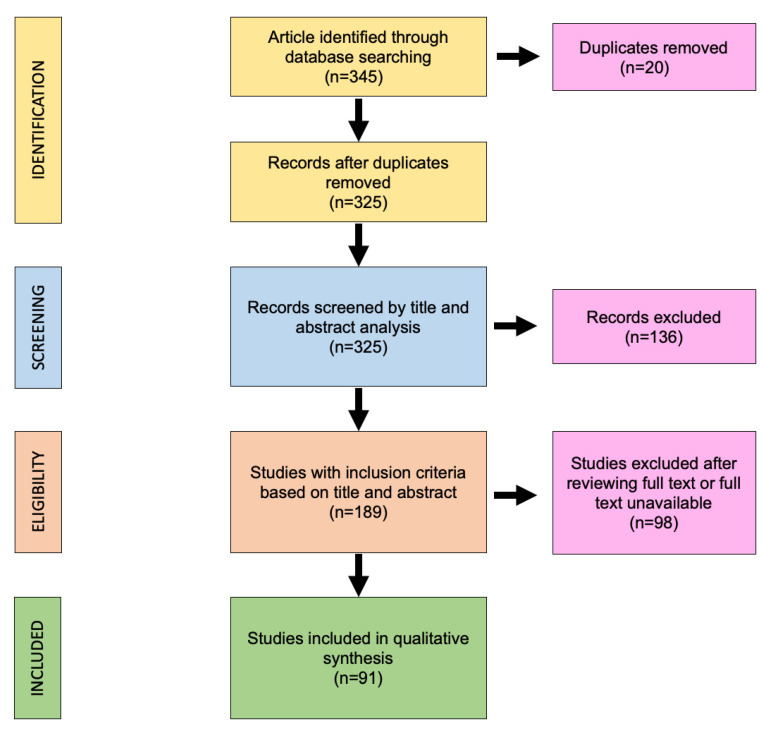
The preferred reporting items for systematic reviews and meta-analyses (PRISMA) diagram followed in this review. The diagram shows the information flow through the different phases of the review and illustrates the number of records that were identified and included.

The search algorithm and review results are outlined in Figure 1. The initial search identified 345 studies, 325 of which remained after removing duplicates. After title and abstract analysis, 136 results were excluded; after the application of our inclusion criteria, a total of 91 studies were included in the systematic review.

**Figure 2:**
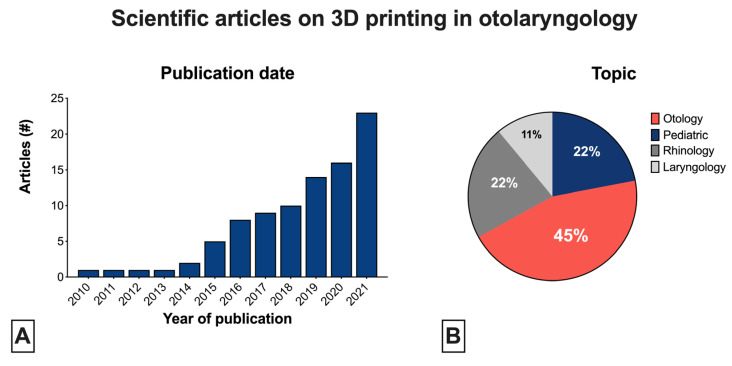
Scientific articles published on 3D printing in the otolaryngology field: (**A**) number of articles sorted by year of publication (range 2010–2021); (**B**) topics of articles.

Studies were published in the range of 2010–2021. We found an increase of the number of 3DP articles per year over time (Figure 2A). The included studies were heterogenous and involved different specific fields of application and areas of interest, as shown in Figure 2B.

**Figure 3:**
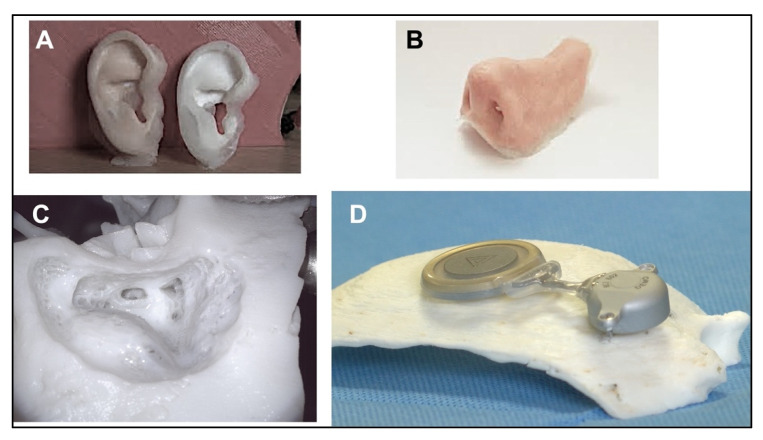
Some examples of 3D uses: (**A**) ear; (**B**) nose; (**C**) temporal bone; (**D**) mastoid bone with bone anchored implant. From Della Volpe et al. [8].

In Figure 3, some examples of 3DP technologies used in otolaryngology are presented.

### 3.1. Otologic Applications

Several studies of 3DP applications in otology focused on surgical training in the field of temporal bone dissection. Thanks to the application of the printed model containing electronic simulators, it was possible to have a real-time alert in case of injury to the noble anatomical structures (nerves, main vessels) that run in the temporal bone during the dissection. Moreover, 3DP systems were suitable for surgical planning and the simulation of possible variants/problems during complex surgery. In 2015, Da Cruz et colleagues showed that 3DP synthetic temporal bones were similar to the human bone in terms of anatomical realism, drill tone, bone dust production, and tactile feedback during dissection [9]. Rose and colleagues created temporal bone models, recreating the bone from the patient’s CT scan; these models were used for real simulation and for the specific training of tympano-mastoidectomy in complicated recurrent cholesteatoma [10,11]. In addition, 3DP systems allow for the reconstruction of the anatomical structure with greater fidelity thanks to the employment of multiple colors and materials reproducing the properties of the trabecular mastoid bone [11].

Bioprinting technology has a high potential in hearing reconstruction, both for partial and total reconstruction of missing parts. In fact, it has been shown that 3DP has good biocompatibility and plasticity and can be used to create patient-specific ossicular to perform ossiculoplasty. Kozin et al. successfully tested a customized 3D-printed prosthesis for repairing bony superior canal defects on a cadaveric temporal bone [12]. Watson et al. showed the utility of using 3DP for ear reconstruction; they treated three patients with missing unilateral ears using auricular protheses with polycaprolactone, helping patients to regain their confidence [13]. In 2016, Kuru et colleagues created the first real-size 3D-printed ear model, with a tympanic membrane and articled ossicles using a micro-CT scan and silicon rubber [14].

Three-dimensional technology can also be used for surgical planning in complicated cases. Mukherjee et al., in 2017, showed the utility of 3D models with an artificial temporal bone for preparation to correct surgical management; the use of this technology improved accuracy during surgery and reduced intraoperative difficulties [15]. In 2021, Della Volpe et al. used 3DP for reproducing a malformed temporal bone of a 5-year-old girl affected by severe atresia auris to identify the better position of a bone anchored hearing implant (BAHI). The patient did not present any negative intra-operatory or post-surgery outcomes and obtained excellent recovery of the hearing function. The authors concluded that 3DP allowed for the best management of the BAHI in case of a severe malformation [8].

### 3.2. Rhinology Applications

Because of the complex and variable anatomy of the nose and paranasal sinuses and the risk of major complications during surgical intervention, rhinology surgery can be a challenge for otolaryngology surgeons. The development of 3D-printed training models for endoscopic sinonasal and skull base surgery could be useful in surgical and preclinical education, as they provide authentic replicas of bony structures.

In 2019, Zhuo and colleagues developed 3D models of nose and paranasal sinuses using patient-specific CT scan data, which had higher fidelity compared to cadaveric dissection [16]. A similar study was performed by Alrasheed, who developed a 3D-printed model of the frontal sinus and osteomeatal complex to use for endoscopic sinus surgical training [17]. In 2016, Muelleman et al. produced personalized 3DP models for preoperative planning in petroclival tumors management [18].

The versability of 3DP systems allows us to fabricate operative templates tailored to the patient’s anatomy, including an osteoplastic flap during frontal surgery or septal prosthesis for large irregular septal perforations [19,20,21]. In 2018, Khan and colleagues performed secondary-close reduction with augmentation rhinoplasty on a 30-year-old-male using a 3D-printed patient-specific silicone implant [19]. Daniel et al. used frontal sinus osteoplastic flaps in a series of 10 patients; flaps were designed from patients’ CT data with a 1–2 mm accuracy. A 3DP poletheretherketone implant was used for the facial reconstruction of a child with craniofacial fibrous dysplasia and had successfully functional and aesthetic results [20]. Onerci Altunay et al., in 2016, developed 3D-printed nasal septal protheses exactly of the size and shape of a patient’s septal defect [21]. In 2021, Mallon and Farnan used a 3D-printed patient-specific skull model of inverted papilloma polyp to perform surgical planning [22]. In the same year, Gillett and colleagues successfully created 3D patient-specific models of pituitary tumors from PET/CT and MRI using four different 3DP techniques to optimize the visualization of the tumor [23].

### 3.3. Laryngology Applications

Laryngeal carcinoma represents the second most common airway cancer in the USA, with a mortality near 25%. Considering the complexity to regenerate a dynamic organ such as the larynx, the application of 3DP on laryngology mainly focuses on tissue repair, on surgical training, and on patients’ information [24,25,26]. In the past years, several attempts to create medical devices that could promote vocal fold regeneration were performed, and 3DP can create, starting from CT images, devices that can be used in surgical practice for surgical training and pre-intervention planning for complex surgical procedures [27,28,29,30]. Three-dimensional-printed models can also help to demonstrate procedures to the patients and surgical team before surgery [31,32,33].

In 2017, Richard and colleagues used CT scan data to create 3D models of two cases of subglottic stenosis. The models were used for surgical simulation and patient education [34]. With the same purpose, Kavanagh et al. created 3D laryngeal models that mimicked natural human tissue for surgical simulation [35]. Grolman et al. created a laryngeal model to simulate the trans-cervical injection of vocal folds and developed a pediatric laryngeal model reproducing several surgical conditions such as subglottic cysts, laryngomalacia, subglottic stenosis, and laryngeal clefts [36].

### 3.4. Pediatric Applications

Interest in 3DP in pediatric otolaryngology is rapidly gaining, especially for the management of airways problems. Bioprinting was successfully used in the creation of patient-specific devices for the treatment of tracheobroncomalacia (TBM) [37]. In 2019, Les et al. developed a bioresorbable 3D-printed tracheal splint to treat a patient with severe TBM [38]. A few years before, Zopf et al. used a polycaprolactone (PCL) scaffold to treat an 8-week-old infant with TBM [39]. In 2014, Morrison et al. employed a personalized 3DP external airway scaffold to treat three children (3, 5, and 16 months) with severe TBM. All patients showed the resolution of respiratory complications over time (follow-up studies up to 30 months) [40].

More recently, 3DP was utilized to facilitate the management of complex upper airway anomalies in newborns. In addition, this technology was utilized to create a pediatric temporal bone model of petrous apex cholesterol granuloma to optimize the operative procedure of endoscopic permeatal drainage of the lesion [41].

In 2020, Stramiello and colleagues performed a systemic review to identify the role of 3DP in pediatric airway obstruction. They identified forty-two articles from 2009 to 2019; 26 of them were classified as discussing direct interventions, while 16 articles were identified as pertaining to surgical planning. Within the first group, only five articles reported direct interventions in human cases, while 18 articles described intervention in models. For direct interventions, the most common material used was polycaprolactone [42].

## 4. Discussion

The aim of this review was to describe the state-of-the-art available clinical applications of 3DP across different otolaryngology areas.

Several opportunities are offered by 3DP systems in modern medicine, despite preliminary evidence. Since the 1990s, hundreds of scientific articles discussed the advantages of 3D printers, and the customization of surgical planning was one of the most important fields of investigation. The creation of anatomical printed models before surgery allowed for the understanding of specific anomalies and guidance for the operative strategy.

In the otolaryngology field, 3DP technology is certainly promising, being increasingly employed from pre-operative planning to printing patient-specific implants and for training and informative purposes. In 2021, Mallon and Farnan reported the first case in Northern Ireland in which the use of a 3D-printed skull model, created from a CT scan, modified the surgical option in a 39-year-old patient with an inverted papilloma polyp [22]. The first explored clinical application was the resection and reconstruction of oromandibular tumors due to their easier medical image processing in comparison to other fields. Similar approaches were employed for complex cases of temporal bone and sinonasal surgery. In 2021, della Volpe et al. used 3DP to simulate the exact position of BAHI in a child with a severe atresia auris, in which the surgical landmarks were totally absent, exposing the patient to a high risk of severe intra, peri, and post-operative complications [8]. Moreover, in this case, the incorrect position of BAHI could make the surgery not useful in terms of functional recovery. The simulation by 3DP allowed them to identify the correct landmarks, helping the surgery to be safely conducted, and the patient obtained excellent hearing recovery. Another 3DP application is its use for surgical and preclinical education, usually made by physical models, animals, or human cadavers. Three-dimensional--printed models were used to train students and residents in complex anatomy and to simulate critical surgical procedures.

The evolution of 3DP models and materials has enabled the reproduction of the finest chromatic details and mechanical properties, resulting in highly representative 3DP simulators; unfortunately, these are still expensive and less employed in the production of didactive devices. The most recent application of 3DP systems is tissue engineering and implantable prostheses, and preliminary data provide encouraging results in terms of safety and effectiveness, opening new frontiers of investigation [43,44]. Morrison and colleagues developed and implanted patient-specific 3DP external airway scaffolds to treat tracheobronchomalacia in three infants, without any graft rejection [40]. In 2019, VanKoevering et al. first demonstrated the potential utility of 3DP technologies in the management of prenatal complex airway anomalies. Starting with fetal magnetic resonance imaging, the authors created a 3D-printed patient-specific model of the craniofacial skeleton of the fetus that showed a maxillofacial mass not extended into the oral cavity [45].

### Limitations of the Study

The main limitation of this study is that we only included articles that reported the clinical application of 3DP technology; therefore, they may not represent the total number of 3DP publications in otolaryngology.

## 5. Conclusions

Three-dimensional-printing technology is increasingly being employed in otorhinolaryngology practice, from pre-operative planning to printing patient-specific implants and for training and informative purposes. This technology seems to be very promising, especially if used for recreating missing structures due to malformations, following oncologic conditions, or after demolitive surgery. However, available evidence is still limited, and additional case-control studies and longitudinal long-term analyses are necessary to fully understand the benefits of this technology compared to auto/allografts and the long-term outcomes.

## Data Availability

Not applicable.
